# Wired to Be Social: The Ontogeny of Human Interaction

**DOI:** 10.1371/journal.pone.0013199

**Published:** 2010-10-07

**Authors:** Umberto Castiello, Cristina Becchio, Stefania Zoia, Cristian Nelini, Luisa Sartori, Laura Blason, Giuseppina D'Ottavio, Maria Bulgheroni, Vittorio Gallese

**Affiliations:** 1 Department of General Psychology, University of Padova, Padova, Italy; 2 Department of Psychology, Centre for Cognitive Science, University of Turin, Turin, Italy; 3 Child Neurology and Psychiatry Unit, Department of Paediatrics, Institute of Child Health IRCCS, Burlo Garofolo, Trieste, Italy; 4 Unit for Prenatal Diagnosis, Department of Obstetrics and Gynaecology, Institute of Child Health IRCCS, Burlo Garofolo, Trieste, Italy; 5 Department of Neuroscience, University of Parma and IIT (Italian Institute of Technology), Section of Parma, Parma, Italy; University of Minnesota, United States of America

## Abstract

**Background:**

Newborns come into the world wired to socially interact. Is a propensity to socially oriented action already present *before* birth? Twin pregnancies provide a unique opportunity to investigate the social pre-wiring hypothesis. Although various types of inter-twins contact have been demonstrated starting from the 11^th^ week of gestation, no study has so far investigated the critical question whether intra-pair contact is the result of motor planning rather then the accidental outcome of spatial proximity.

**Methodology/Principal Findings:**

Kinematic profiles of movements in five pairs of twin foetuses were studied by using four-dimensional ultrasonography during two separate recording sessions carried out at the 14^th^ and 18^th^ week of gestation. We demonstrate that by the 14th week of gestation twin foetuses do not only display movements directed towards the uterine wall and self-directed movements, but also movements specifically aimed at the co-twin, the proportion of which increases between the 14^th^ and 18^th^ gestational week. Kinematic analysis revealed that movement duration was longer and deceleration time was prolonged for other-directed movements compared to movements directed towards the uterine wall. Similar kinematic profiles were observed for movements directed towards the co-twin and self-directed movements aimed at the eye-region, i.e. the most delicate region of the body.

**Conclusions/Significance:**

We conclude that performance of movements towards the co-twin is not accidental: already starting from the 14th week of gestation twin foetuses execute movements specifically aimed at the co-twin.

## Introduction

One-to-one interactions are the cradle of social cognition. Infants do not develop social understanding by merely watching other people at a distance. Rather, they learn by engaging in reciprocal exchanges with others [1]–[7]. Even hours after birth, newborns have been found to show preparedness for social interaction that, among other things, is expressed in their imitation of facial gestures [8], [9]. Altogether such evidence indicates that newborns come into the world wired to socially interact. But, is a propensity to interact with others demonstrable *before* birth?

Twin pregnancies provide a unique opportunity to investigate the social pre-wiring hypothesis. Unlike ordinary siblings, twins share a most important environment – the uterus. If a predisposition towards social interaction is present before birth, one may expect twin foetuses to engage in some form of interaction. Although inter-twin contact has been demonstrated starting from the 11^th^ week of gestation [10], no study has so far investigated the critical question of whether twin foetuses plan and execute movements directed towards each other. Put differently, whether intra-pair contact is the result of motor planning rather then the accidental outcome of spatial proximity. Whilst twins are initially too distant and their movements too weak to reach one another, with advancing gestational age contact between them becomes possible and soon almost inevitable. From the 11^th^ week onwards, different patterns of inter-twin contact such as head to head, head to arm and arm to head contact are observed [10]. It is, however, between the 15^th^ and 22^nd^ week that intra-pair contact becomes a constant and increasing feature of all twin pregnancies [11]–[13].

Whereas inter-twin contact is well established, little is known about the organization of movements bringing twins in touch. The motor behaviour of foetuses has traditionally been described in terms of reflexes rather than actions [14]. Although reflexes serve important functions, they are stereotyped, elicited and once launched run their predetermined course. This signifies, for instance, that reflexes are not goal directed, are not subject to learning and do not adjust to future states in a prospective fashion [14]. In contrast with the idea that foetuses only display reflexes, Zoia and colleagues [15] recently demonstrated kinematic adaptation to the somatosensory properties of the target in 22-week-old single foetuses. Three types of hand movements were isolated and subsequently analyzed: movements ending at contact of fingers with the mouth, movements ending at contact of fingers with the eye, and movements directed away from the body, towards the uterine wall. The results showed that the spatial and temporal characteristics of foetal movements were by no means uncoordinated, but depended on the goal of the different motor acts, suggesting a surprisingly advanced level of motor planning.

Along these lines it might be advanced that, if foetuses plan movements towards the co-twin, then a specific kinematic pattern related to the social end goal of the movement might be expected. Social actions differ from those used in negotiating the physical environment in many important aspects. The fact that one's own actions affect the behaviour of the person towards whom they are directed creates new action problems, which are not encountered when the actions are directed towards objects [14], [16]. In adults, indeed, specific kinematic profiles have been shown to differentiate social actions from actions performed in isolation [17]–[19]. In particular, kinematics for arm-actions aimed at a social target have shown to be different from those of similar movements ending on a physical object [17]. If inter-twin contact reflects motor planning, then differences in kinematics might be expected between movements directed towards the co-twin and movements directed towards one's own body or the uterine wall. Here we tested this hypothesis by investigating the kinematics of movement in five pairs of twin foetuses. Arm movements were studied using four-dimensional ultrasonography (4D-US) during two separate recording sessions carried out at the 14^th^ and 18^th^ week of gestation. Foetuses were videotaped for 20 minutes in each session and the video recordings were then digitized with purposely-developed software for off-line kinematic analysis. Three main categories of arm movements were isolated and subsequently analyzed: i) self-directed movements, including hand to mouth and hand to eye movements; ii) non-targeted movements, encompassing movements directed towards the uterine wall; and iii) other-directed movements, including hand to the back and hand to the head of the co-twin. We employed three analyses in order to explore whether the organization of foetal movements differed depending on the nature of the executed movement. The first analysis compared the incidence of each type of movement at the two gestational periods. The trend in the incidence of motor activities is considered to directly reflect developmental and maturational processes of the foetal central nervous system [20]–[22]. If specific movement patterns underlie other-directed movements, the trend in the incidence of other-directed movements might be expected to be dissociated from that of movements directed towards one's own body or outer-directed movements. The second analysis compared the kinematic profiles of the different categories of movements. Based on the social pre-wiring hypothesis, we predicted that the kinematic pattern of other-directed movements would be different from the kinematic pattern of those movements directed towards one's own body or outer-directed movements. Finally, the third analysis employed a comparison of self-directed movements towards the mouth and the eye region, and movements directed towards the sibling. Kinematic adaptation to the properties of the target has been reported in single foetuses by the 22^nd^ week of gestation [15]. Because the presence of a co-twin may facilitate or prime an anticipated propensity to act, signs of kinematic differentiation between hand to mouth and hand to eye movements might be expected to appear earlier in twin foetuses.

## Methods

### Ethic statement

The experimental procedures were approved by the Institutional Review Board at the University of Trieste, and were in accordance with the Declaration of Helsinki (Sixth revision, 2008). All mothers participating in the study gave their informed written consent to participate in the study.

### Participants

The five women with a twin pregnancy who participated in this study were a convenient sample of low-risk pregnant women attending the Institute of Child Health I.R.C.C.S. Burlo Garofolo (see Table 1 and Table 2). Prior to undergoing the 4D-US test, all participants were asked to attend a meeting in which the experimental procedures were explained. The designation of “low risk” for foetuses was made during the initial obstetric appointment based on maternal medical history and checked at each subsequent examination by the gynaecologist.

**Table 1:** Characteristics of the mothers involved in the study.

Mothers	Age	Education	SES	BMI	Smoker	BP	AF	Dating	Delivery
1	31	Junior High School	hairdresser	20.28	stopped smoking	110/70	Normal	US	Caesarean
2	30	Junior High School	store clerk	23.05	stopped smoking	135/85	Normal	US	Caesarean
3	20	Junior High School	unemployed	20.82	No	110/70	Normal	US	Caesarean
4	29	High School	store clerk	26.07	No	130/95	Normal	US	Caesarean
5	32	High School	teacher	19.19	No	105/65	Normal	US	spontaneous

Notes. SES social economical status, BMI body mass index, BP blood pressure, AF amniotic fluid.

**Table 2:** Birth data for the five twin-foetuses.

Foetuses	BGA	Sex	Apgarfirst minute	Apgarfifth minute	BW (g)	pH	BE	NE	F. Pos	Placenta	NICU
A. - F.	34+6	f/f	9 - 9	10 - 10	2070–2020	7,36 - 7,32	−1,5; −1,2	normal	pod - cef	anterior	yes
L. - T.	36+6	m/m	9 - 9	10 - 10	2980–2820	7,24 - 7,24	−2,6; −4,3	normal	cef - pod	anterior	no
A. - A.	35+1	f/f	8 - 7	10 - 10	2190–2290	7,27 - 7,27	−4,5; −3,9	normal	cef - cef	anterior	yes
G. - S.	36+4	f/m	9 - 8	10 - 9	2600–2970	7,18 - 7,15	−10,6; −12,0	normal	cef - cef	ant - post	yes
B. - S.	33+3	f/m	7 - 8	8 - 9	1860–1870	7,36 - 7,22	−0,0; −8,7	normal	cef - pod	ant - post	yes

Notes: BGA Birth Gestational Age, BW birth weight, BE base excess, NE neurological examination, F. Pos foetal position, NICU neonatal intensive care unit.

### Instrumentation

For the purpose of this study we analyzed the abdominal four-dimensional ultrasound (that is 3D images in time known as 4D-US; Voluson 730 Expert by GE Medical Systems) of ten foetuses. The 4D-US examination was conducted according to previous studies [21]. The ultrasound technique allows changes to be made to several parameters: depth of the visual field, the sweeping angle that defines the sample volume and the frame rate. These parameters are directly related to each other. In this study the machine was set at the fixed frame rate of 4 Hz, to guarantee the same number of images per second. The crystal array of the transducer swept mechanically over the volume of the uterine cavity, framing the defined regions of interest (ROI). To visualize the foetal movements the transducer, which was maintained stationary, was positioned so that a frontal view of the foetus, including head, arms, hands, thorax and abdomen was obtained. Foetuses were taped for 20 minutes. The video recordings were then digitized through our purposely developed software which allows off-line kinematic analysis for hand movements (see ‘kinematic analysis’ section below).

### Procedure

Each woman was identified by the prenatal sonologist during her first visit at the 12^th^ week of pregnancy and foetal age was calculated comparing the mother's last menstruation date and the measurements of the foetus (Crown Rump Length) taken during the ultrasound examination. At the time the couple agreed to take part in this study, the appointment was made for the first ultrasound imaging session during the 14^th^ week. The following appointment was within the 18^th^ week. The tests were conducted in a quiet room, with the women lying on their backs. Each examination was conducted in the early afternoon, two hours after lunch. Each observation period lasted 20 minutes and was video recorded. At the end of each video recording the humeral length was measured and later used in the kinematic analysis. The images were obtained with the expectant mother in a semi-recumbent position, with diminished light, consistent with clinical obstetrical imaging. Each woman was interviewed prior to ultrasound imaging to record any environmental changes in work or family conditions (i.e., stress that could possibly affect the movements of the foetuses) that she may have perceived over the preceding 4 weeks. She was also asked to complete two questionnaires involving both perceived state anxiety and trait anxiety, which always resulted within the normal range.

### Type and occurrence of movements

**Figure 1:**
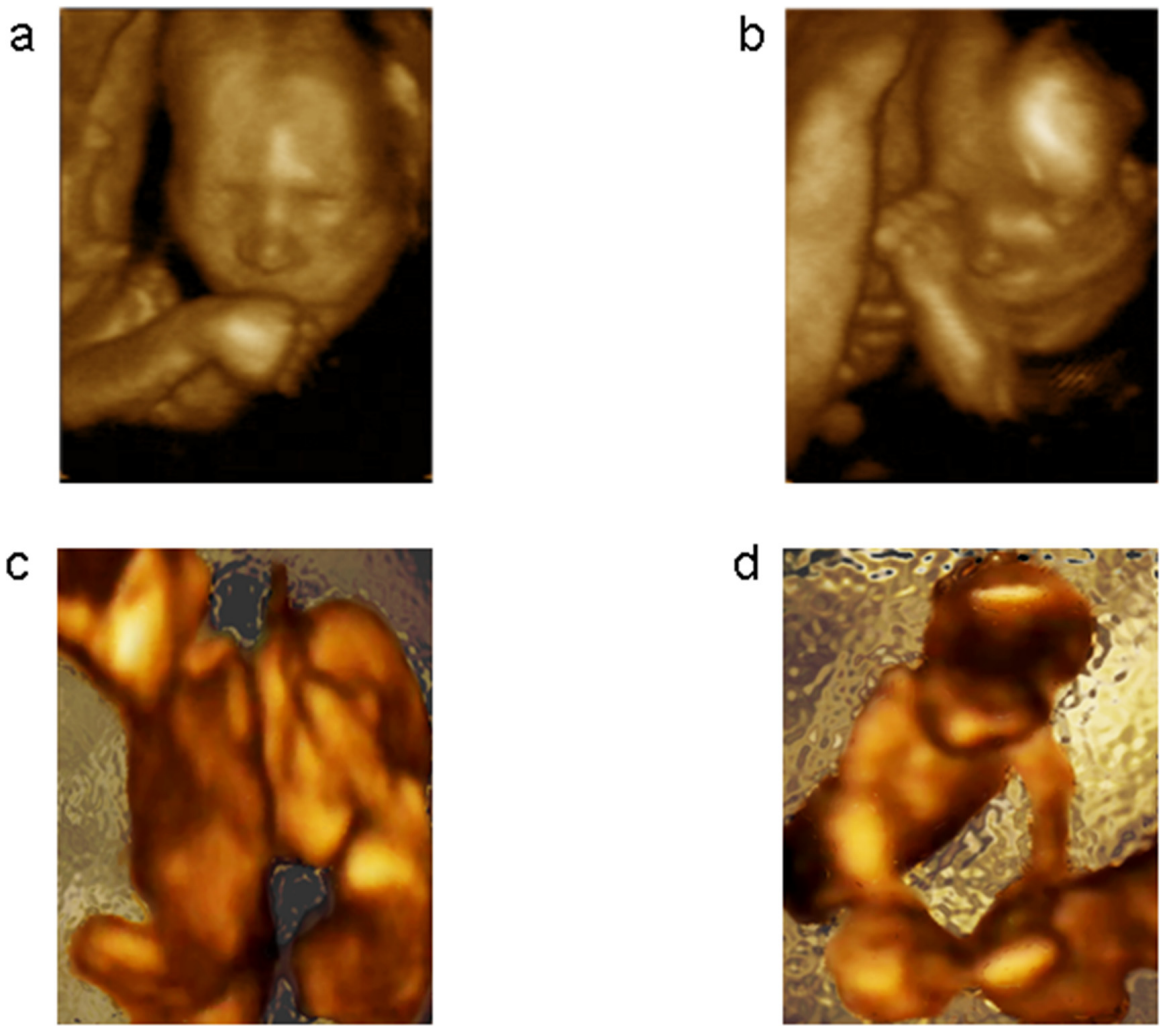
Types of movements. a, Video frame representing a self-directed movement towards the mouth. b, Video frame representing a self-directed movement towards the eye. c, Video frame representing the foetus reaching towards and “caressing” the back of the sibling. d, Video frame representing the foetus reaching towards and “caressing” the head of the sibling.

Two expert judges, unaware of the study rationale and blind to the experimental conditions, assessed all the recordings for each foetus. Five types of arm movements were isolated and subsequently analyzed: i) hand to mouth self-directed movements, when hand movements ended at contact of fingers with the mouth (Fig.1a); ii) hand to eye self-directed movements, when movements ended at contact of fingers with the eye region (Fig. 1b); iii) non-target outer-directed movements, when movements were directed away from the body towards the uterine wall; iv) other-directed movements ending with hand to the back of the sibling (Fig.1c); v) other-directed movements ending with hand to the head of the sibling (Fig. 1d). Reliability between the two judges was very high (Cohen's κ = .93).

It has been shown that in pathological pregnancy the movement of the foetus can be influenced by amniotic fluid volume [23]. Although in our studies we included only healthy pregnancies, we checked that the estimate of amniotic fluid was within the normal range (see Table 2). While underlining that the combined results of the right and left hand movements are presented in this article, issues concerned with asymmetries are beyond the scope of this work. No significant difference was observed between right and left-handedness for the total of movements (Wilkoxon signed-rank test, Z = 0.17, *ns*). Further, because it has been observed that the number of movements might be greater among females than males at around the 16^th^–20^th^ week of gestation, we investigated the possible differences with regard to sex. Of the five pregnant women, two gave birth to twins which were both female, one to twins which were both male and two to twins which were one female and one male. No significant differences were observed with regard to sex (Mann-Whitney test: Z = 0.34, *ns*).

### Kinematic Analysis

**Figure 2:**
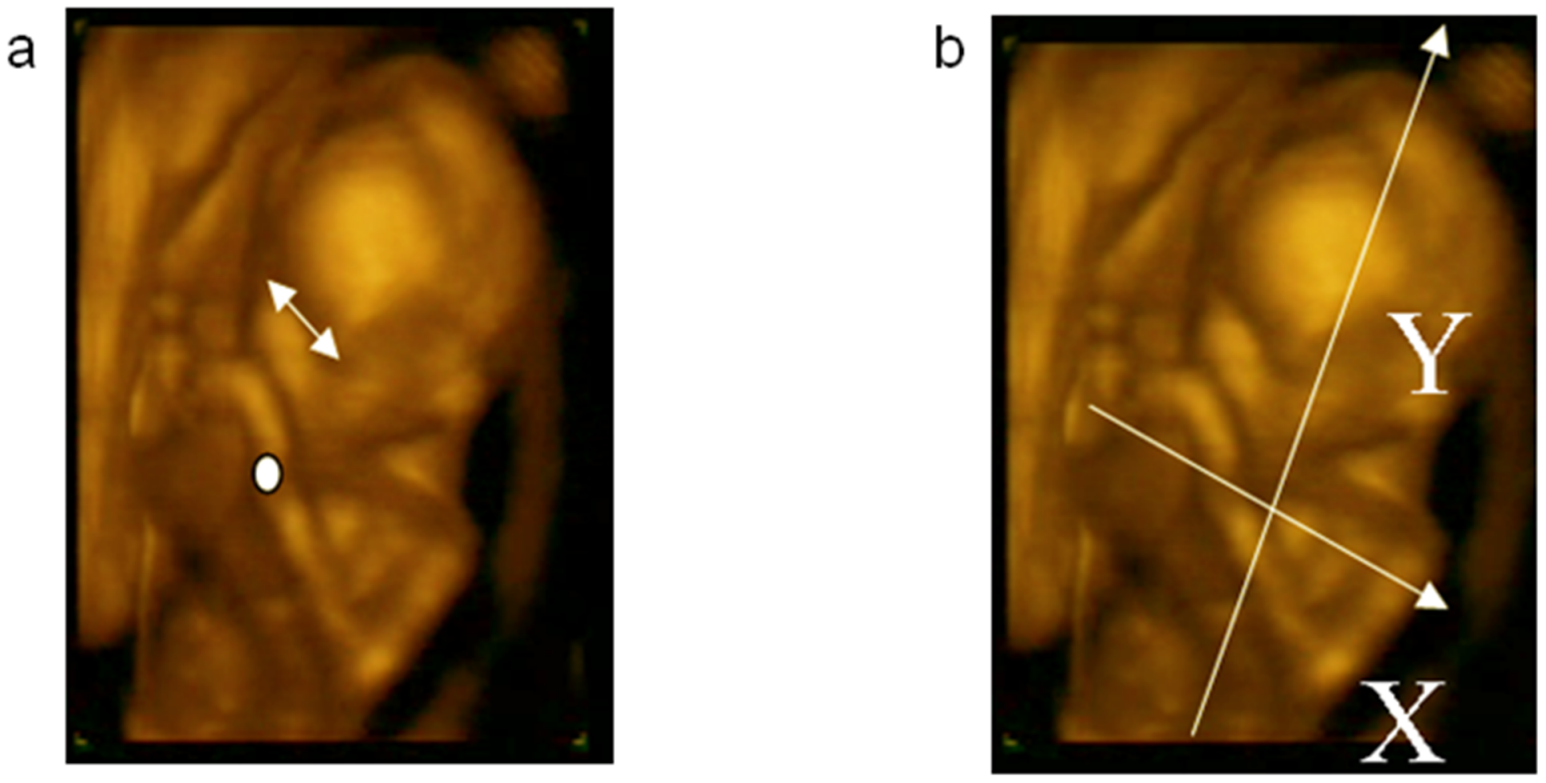
Frame of reference and measurement unit used to refer the examined body position. a, Intraocular distance and position of the wrist marker. b, Axes used to perform the 2D kinematic analysis.

Kinematic analysis of foetuses' spontaneous and unskilled upper limb movements presents formidable problems. First and most importantly, we had to consider the obvious “lack of co-operation” by the subjects performing interesting movements: foetuses do not act on “start” and “end” commands and an unfavourable foetal position may prevent visualization of the start and end point. Second, whereas in normal conditions anatomical landmarks can be referred to absolute co-ordinates, the 4D-US technique does not enable the definition of an absolute frame of reference. Because the field of view of the transducer is continuously changing, performed movements need to be referred to a relative frame of reference. In the present study, we considered a “foetus-centred” co-ordinates frame whose origin coincided with the mid-point between the shoulders of the foetus. Third, because anthropometric parameters change from one foetus to the other and within the same foetus at different gestational weeks, length has to be defined by using a relative measurement unit, instead of an absolute measurement unit (e.g. millimetres). As intra-ocular distance is intimately related to head size [24] and head size is commonly used to identify gestational age [25], we adopted intra-ocular distance as the measurement unit. This procedure allowed us to compare the amplitude of the movements for different gestational ages. Finally, although the 4D-US technique enables 3D visualization of the foetus, kinematic analysis has to be in 2D. This is because at present 4D scanning supplies only a 2D movie of the 3D acquisition and does not provide digital 3D co-ordinates. Thus, in order to capture the dynamics of spontaneous movements, we imported the video recordings for foetuses' movements into in-house software developed to perform two-dimensional (2D) kinematic analysis. Movements were discarded from the analysis if one of the following conditions occurred: the foetus was not in a supine position or was not clearly visible from the starting to the end point or if the head was turned so that the eye position was not available. As it was not possible to ask the foetus to start from a precise location or at a specific command, the criterion for hand movements to begin was when the hand was stationary within the chest area (below the shoulders and above the belly). The criterion for ‘touched target’ was when the hand clearly stopped on the mouth, eye, back of the sibling, head of the sibling areas. We took great care to discern target touch from proximity. Velocity change from zero was the threshold criteria for determining the start of the movement. On the basis of the above criteria only 58% of the recorded movements were actually considered for kinematic analysis. Each of the identified movements was classified taking into account the starting and ending area. The next step was to assign the marker on the foetus's arm at wrist level (Fig. 2a) and to track it frame by frame (frame duration: 250 milliseconds) for the entire movement, with respect to the target zone (eye and mouth). The wrist marker was used to compute arm velocity (displacement derivative) data. This procedure was performed manually and post-hoc by the same analyst for all foetuses. Finally, the movement was reconstructed considering the mid-point between the shoulders as the frame of origin and the line joining the shoulders as the horizontal axis (Fig. 2b). The vertical axis was computed as the perpendicular of the horizontal axis given that kinematic analysis was performed in two dimensions. For the velocity profiles the spatial measurement unit was the intra-ocular distance. Thus a unit measure of 1 does not refer to “1 millimetre” but to “1 intra-ocular distance”. As a consequence, the values we obtained are only meaningful within the subset of the analyzed foetuses.

## Results

### Incidence of each type of movement at 14 and 18 weeks

**Figure 3:**
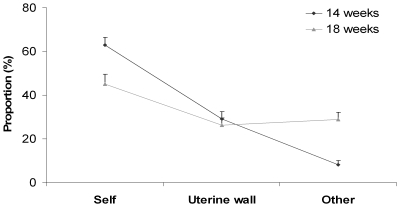
Schematic representation of the analysis considering the proportion of each type of movement. Proportion of movements performed by the foetuses towards the self, outer-directed movements and movements towards the co-twin at the 14^th^ - (solid line) and 18^th^ – (dashed line) week of gestation. Error bars represent the standard errors of the means.

The first analysis compared the incidence for each type of movement at the two gestational periods considered. Frequency of occurrence for each type of movement at the two gestational periods is reported in Table 3. Whereas no differences were revealed when comparing the proportion of movements performed towards the uterine wall at 14 and at 18 weeks [*t*_(9)_ = .859; *P*>0.05], the proportion of self-directed movements was greater at 14 than at 18 weeks [*t*_(9)_ = .480; *P*<.007; see Fig. 3]. By contrast, for other-directed movements the proportion was greater at 18 than at 14 weeks [*t*_(9)_ = −5.940; *P*<.0001].

**Table 3:** Number of movements recorded for each of the foetuses considered.

			Individual movements			Social movements	
Mothers	Foetuses	Weeks	Mouth	Eye	Outer	Back of the sibling	Head of the sibling
MM	1	14	12	10	8	4	2
		18	10	11	12	8	7
	2	14	9	6	9	0	1
		18	5	5	5	6	4
BS	1	14	20	12	10	2	3
		18	10	8	15	7	8
	2	14	29	9	17	5	5
		18	15	11	10	9	8
CD	1	14	8	0	4	1	0
		18	2	0	10	7	4
	2	14	20	16	4	0	1
		18	8	7	2	3	5
FA	1	14	5	7	10	2	1
		18	9	8	8	4	3
	2	14	1	8	9	0	0
		18	1	8	7	1	2
MB	1	14	13	6	7	1	0
		18	7	8	6	3	2
	2	14	15	6	9	2	1
		18	9	6	9	3	2

### Movement duration and velocity profiles for the different types of movement

The dependent variables that were thought to be specifically relevant to the scientific hypotheses being tested were movement duration and deceleration time. Movement duration was calculated as the time between the beginning and the end of the movement. Deceleration time was calculated as the time from peak velocity to the end of the movement. These variables were chosen because consistent results within the reaching literature have shown that movement duration and deceleration time are dependent upon the accuracy level dictated by the target [26] and by the context within which a movement occurs (individual vs. social) [17].

**Figure 4:**
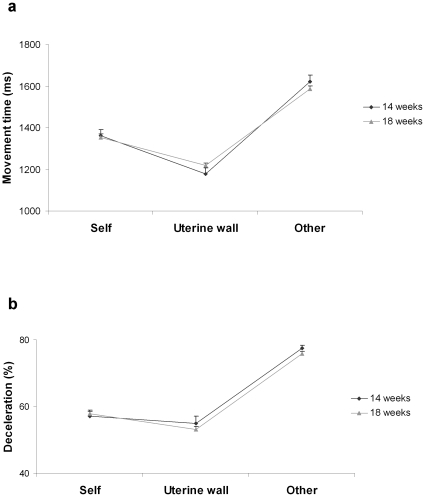
Kinematic profiles for different types of movement at the 14^th^ and 18^th^ week of gestation. Movement duration and deceleration time for self-directed movements, non-target movements and social movements at the 14^th^ and 18^th^ week of gestation. a, Movement duration was longer for arm movements performed towards the sibling than for movements performed towards the self and outer-directed movements both at the 14^th^ and at the 18^th^ week of gestation. b, Independently of the period of gestation, the percentage of time spent decelerating was greater for movements performed towards the sibling than for those performed towards the self and outer-directed movements. Error bars represent the standard errors of the means.

**Figure 5:**
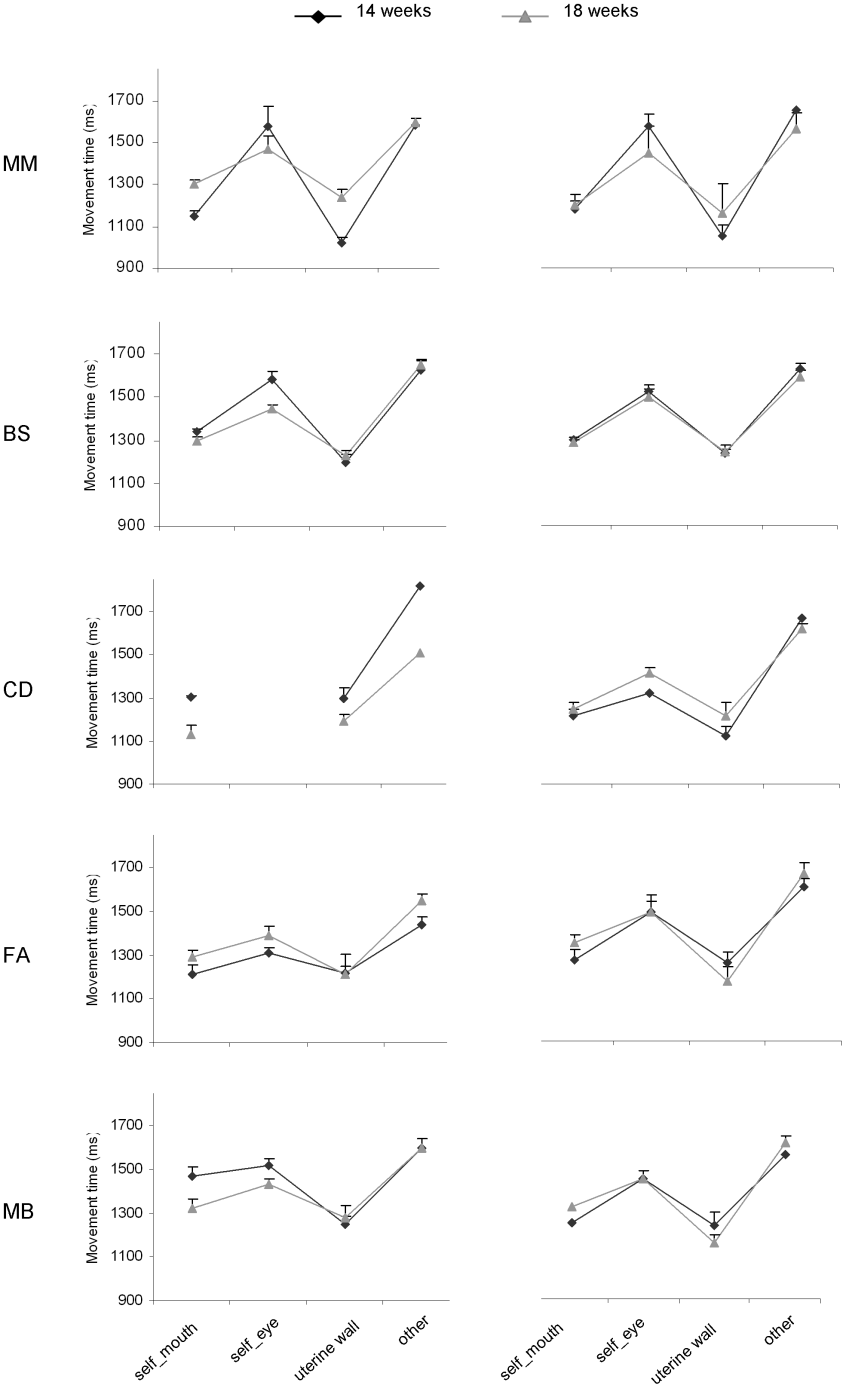
Movement duration for different types of movement at the 14^th^ and 18^th^ week of gestation for each foetus.

**Figure 6:**
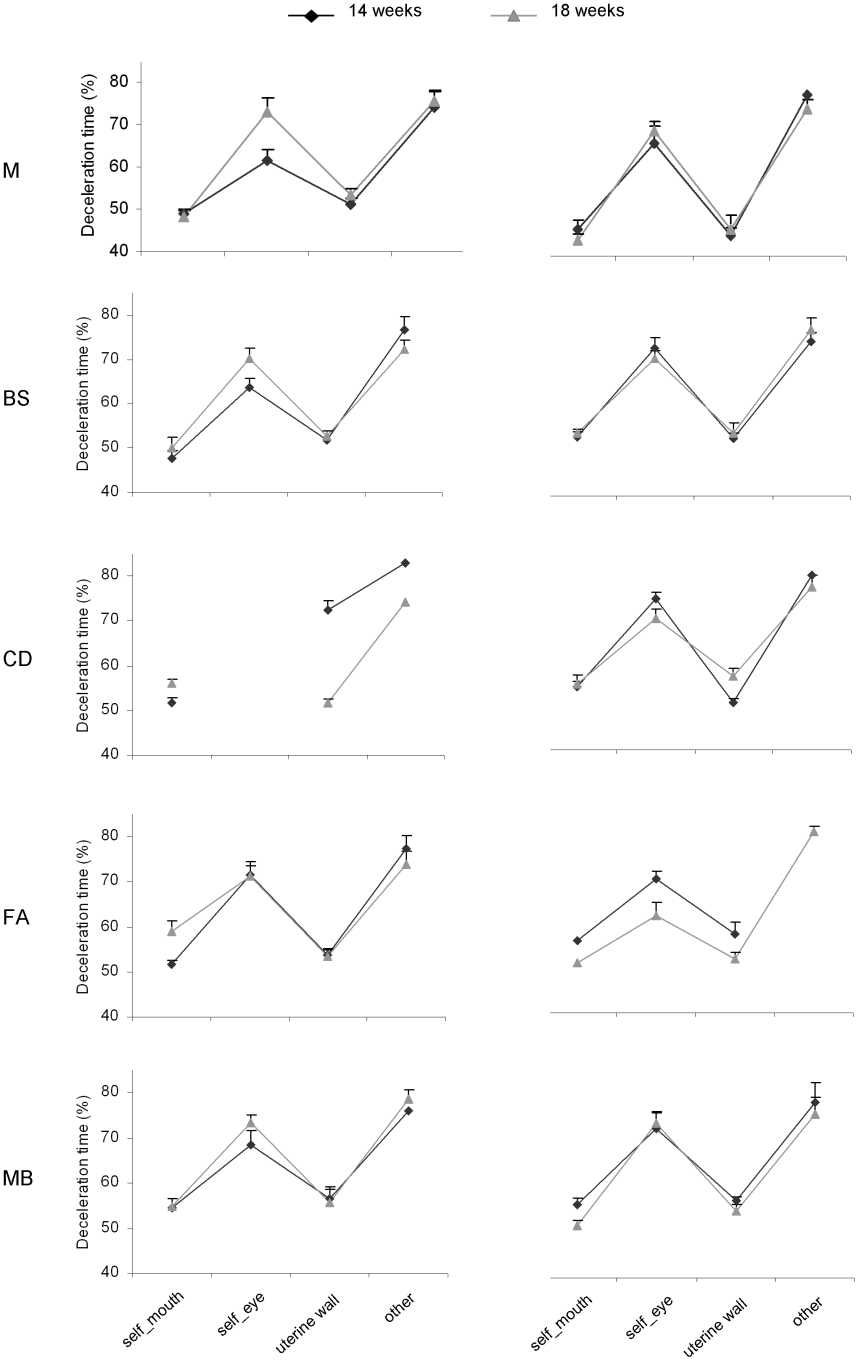
Deceleration time for different types of movement at the 14^th^ and 18^th^ week of gestation for each foetus.

To compare movement duration and deceleration time for the different types of movements we performed a repeated measure analysis of variance with gestational week (14^th^ vs. 18^th^) and type of movement (uterine wall, self-directed, other-directed) as within-subjects factors. We observed a significant increase in movement duration [F_(2,18)_ = 145.32, *P*<0.001] and deceleration time [F_(2,18)_ = 153.630, *P*<0.001] depending on the type of movement performed. Movement duration was longer for other-directed movements than for movements towards either the self or the uterine wall (*P*_s_<0.001; Fig. 4a, Fig. 5). It was also longer for self-directed movements than for outer-directed movements (*P*<0.05). Similarly, deceleration time was greater for other-directed than for either self-directed or outer-directed movements (Fig. 4b; *P*_s_<0.001). Finally, self-directed movements showed a longer deceleration phase than those performed towards the uterine wall (*P*_s_<0.05; Fig. 4b; Fig. 6). For both measures the main effect of ‘gestational week’ and the interaction ‘gestational week’ by ‘type of movement’ were not significant (*P*_s_>0.05), suggesting that the reported kinematic differences across type of movements hold regardless of the gestational period considered.

### Kinematic adaptation to the properties of the target

**Figure 7:**
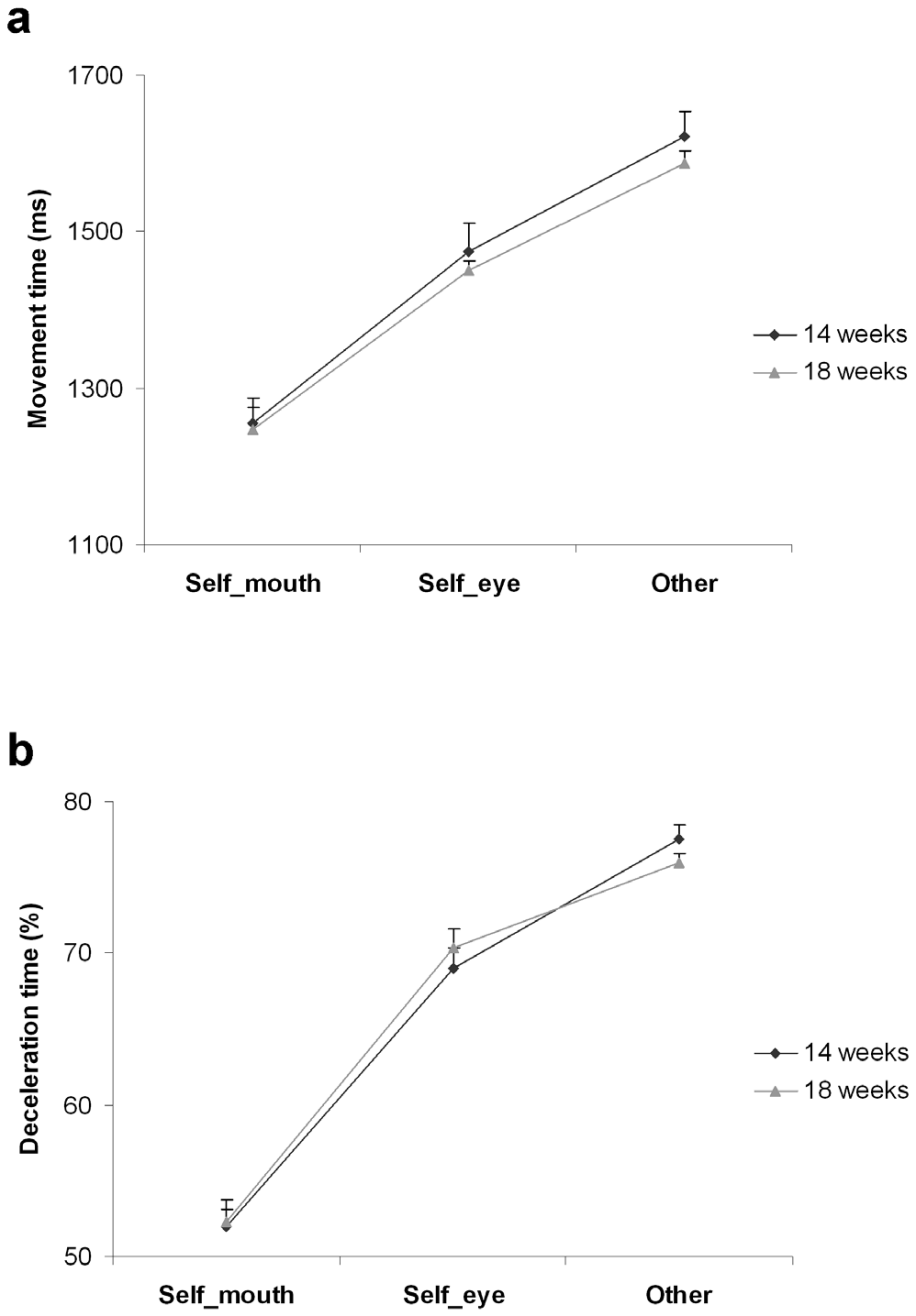
Kinematics adaptation to the properties of the target. Movement duration and deceleration time for self-directed movements towards the mouth and the eye region, and movements directed towards the sibling at the 14^th^ and 18^th^ week of gestation. a, Movement duration for movements performed towards the co-twin was longer than for self-directed movements towards the eye or the mouth. Duration of movements towards the eye region was longer than for those directed towards the mouth. b, The percentage of time spent decelerating was greater for movements towards the co-twin than for movements towards the eye or the mouth. Deceleration time for movements towards the eye region was longer than that for those directed towards the mouth. For both dependent measures the size of the target effect was comparable at the 14^th^ and 18^th^ week. Error bars represent the standard error of the means.

To explore how kinematics adapted to the properties of the target we performed an ANOVA with gestational week (14^th^ vs. 18^th^) and target (mouth, eyes, other) as within-subjects factors on both deceleration time and movement duration. We observed a significant main effect of target on both movement duration [F_(2,18)_  = 93.826, *P*<0.001; Fig. 7a] and deceleration time [F_(2,18)_  = 377.726, *P*<0.001; Fig. 7b]. Movement duration for movements performed towards the co-twin was longer than for self-directed movements towards the eye or the mouth (*P*_s_<0.001). Duration of movements towards the eye region was longer than that for those directed towards the mouth (*P*<0.001). A similar pattern was observed for deceleration time, which was prolonged for movements towards the co-twin compared to movements towards the eye or the mouth region. (*P*_s_<0.001). Deceleration time for movements towards the eye region was longer than that for those directed towards the mouth (*P*<0.001). By contrast, for both dependent measures the main effect of gestational week and the interaction gestational week by target were not significant (*P*_s_>0.05), indicating that the effect of target was comparable at 14 and 18 weeks.

## Discussion

Twin pregnancies constitute an experiment of nature which offers the unique opportunity to explore social behaviour before birth. By investigating kinematic profiles of movements in five pairs of twin foetuses, we demonstrated that, by the 14^th^ week of gestation, twin foetuses not only display movements directed towards the uterine wall and self-directed movements, but also movements specifically aimed at the co-twin. Whereas the proportion of self-directed movements decreased between the 14^th^ and the 18^th^ week of gestation and no difference was revealed in the proportion of movements directed towards the uterine wall at the two gestational periods, the incidence of other-directed movements progressively increased to reach 29% of observed movements at 18 weeks. The decrease in the incidence of self-directed movements matched previous reports on the evolution of the spontaneous motor activities in single foetuses: possibly as a result of foetal neurological maturation as well as reduction in intrauterine space, movements of the limbs tend to decrease during the second semester [27]–[29]. Other-directed movements showed a trend in the opposite direction, increasing over the interval considered. This finding is consistent with previous reports assessing inter-twin contact in the second semester: whereas inter-twin contact can be considered a rather exceptional event before the 10^th^ week of gestation [10], the number of contacts between twins rapidly increases during the second semester [11]–[13].

Analysis of the kinematic profiles for the different categories of movements corroborates our main hypothesis that these early contacts do not occur accidentally, but reflect motor planning. In keeping with the social pre-wiring hypothesis, we found that movement duration and deceleration time were longer for other-directed movements than for movements towards the self or the uterine wall. These differences in kinematic profiles were surprisingly consistent across foetuses and held independently of the gestation period considered, suggesting that already starting from the 14^th^ week of gestation intra-pair contact resulted from the planning and performance of social movements obeying specific kinematic patterns.

The ability to scale kinematics depending on the goal of the action was further confirmed by the comparison of kinematic profiles for self-directed movements towards the mouth and the eye region, and movements directed towards the sibling. In singletons evidence of kinematic adaptation to target properties has been provided in 22-week-old foetuses, but not in 14- and 18-week old ones: up to the 18^th^ week of gestation, reaching is rather inaccurate and there is no indication that the eye, the most delicate region of the body, is treated differently from the mouth [15]. Here we found that in twins a differential kinematic pattern for movements performed towards the eye region and movements performed towards the mouth were already evident at the 14^th^ week of gestation. At 14 as well as at 18 weeks, movement duration was longer and deceleration time was more prolonged for movements towards the eye compared to movements towards the mouth. Consistently with available evidence on acceleration of physical and neurological maturity in multiple pregnancies [30], this precocious differentiation of movement patterns might be regarded as an expression of early motor development. Interestingly, the kinematic profile of movements directed towards the co-twin displayed an even higher degree of accuracy: movement duration was longer and the percentage of time spent decelerating was greater for movements directed towards the co-twin than for self-directed movements aimed at the eye or the mouth.

Kinematic adaptation to the properties of the target has been interpreted as evidence that predictive processes might already be operating in foetuses [15], [31]. Foetuses might anticipate the sensory consequences of a movement and use them to plan an action related to the nature of the target. Differential movement patterns observed for hand to eye and hand to mouth movement might thus indicate that information about the different sensations obtained by target organs are used to adjust the approach of the hand [14]. The finding that foetuses treat the co-twin as a special kind of target suggests that in twin pregnancies motor control might extend to incorporate information from intra-pair stimulation.

In this article we describe changes in the kinematic profiles of movement in twin foetuses probing the social dimension of motor planning and control. The central advance of this study is the demonstration that ‘social actions’ are already performed in the second trimester of gestation. Starting from the 14^th^ week of gestation twin foetuses plan and execute movements specifically aimed at the co-twin. These findings force us to predate the emergence of social behaviour: when the context enables it, as in the case of twin foetuses, other-directed actions are not only possible but predominant over self-directed actions. The prenatal ‘social’ interactions described in this paper epitomize the congenital propensity for sociality of primates in general and of humans in particular, grounding for the first time such long-held intuition [32] on quantitative empirical results. Future work will be crucial for elucidating the relation between the foetal origin of social behaviour and normal and abnormal brain development. As foetal behavioural patterns directly reflect developmental and maturational processes of the foetal central nervous system [22], it might be advanced that social patterns might represent early markers of the appearance of developmental disorders affecting the social dimension of behaviour.
